# Preoperative assessment of retroperitoneal Liposarcoma using volume-based ^18^F-FDG PET/CT: implications for surgical strategy and prognosis

**DOI:** 10.1186/s12880-023-01179-z

**Published:** 2023-12-18

**Authors:** Dao-Ning Liu, Jian-Hui Wu, Zhong-Wu Li, Hai-Yue Wang, Xiu-Yun Tian, Chun-Yi Hao

**Affiliations:** 1 Key laboratory of Carcinogenesis and Translational Research (Ministry of Education/Beijing), Department of Hepato-Pancreato-Biliary Surgery/Sarcoma center, Peking University Cancer Hospital & Institute, No. 52 Fucheng Road, Haidian District, Beijing, People’s Republic of China; 2 Key laboratory of Carcinogenesis and Translational Research (Ministry of Education/Beijing), Department of Pathology, Peking University Cancer Hospital & Institute, Beijing, People’s Republic of China

**Keywords:** Metabolic Tumor volume, Retroperitoneal Liposarcoma, ^18^F-FDG PET/CT, Diagnosis, Prognosis

## Abstract

**Purpose:**

Retroperitoneal liposarcoma (RLPS) poses a challenging scenario for surgeons due to its unpredictable biological behavior. Surgery remains the primary curative option for RLPS; however, the need for additional information to guide surgical strategies persists. Volume-based ^18^F-FDG PET/CT may solve this issue.

**Methods:**

We analyzed data from 89 RLPS patients, measuring metabolic tumor volume (MTV), total lesion glycolysis (TLG), and maximum standardized uptake value (SUVmax) and explored their associations with clinical, prognostic, and pathological factors.

**Results:**

MTV, TLG of multifocal and recurrent RLPS were significantly higher than unifocal and primary ones (*P* < 0.001, *P* < 0.001, *P* = 0.003 and *P* = 0.002, respectively). SUVmax correlated with FNCLCC histological grade, mitotic count and Ki-67 index (*P* for G1/G2 = 0.005, *P* for G2/G3 = 0.017, and *P* for G1/G3 = 0.001, *P* < 0.001 and *P* = 0.024, respectively). MTG, TLG and SUVmax of WDLPS were significantly lower than DDLPS and PLPS (*P* for MTV were 0.009 and 0.022, *P* for TLG were 0.028 and 0.048, and *P* for SUVmax were 0.027 and < 0.001, respectively). Multivariable Cox analysis showed that MTV > 457.65 (*P* = 0.025), pathological subtype (*P* = 0.049) and FNCLCC histological grade (*P* = 0.033) were related to overall survival (OS).

**Conclusions:**

Our findings indicate that MTV is an independent prognostic factor for RLPS, while MTV, TLG, and SUVmax can preoperatively predict multifocal lesions, histological grade, and pathological subtype. Volume-based ^18^F-FDG PET/CT offers valuable information to aid in the decision-making process for RLPS surgical strategies.

**Supplementary Information:**

The online version contains supplementary material available at 10.1186/s12880-023-01179-z.

## Introduction

Liposarcoma constitutes the majority of retroperitoneal sarcomas, encompassing well-differentiated liposarcoma (WDLPS), dedifferentiated liposarcoma (DDLPS), and pleomorphic liposarcoma (PLPS) [1]. Currently, surgical resection serves as the only curative approach for retroperitoneal liposarcoma (RLPS) [2]. However, surgeons require additional information to formulate a reliable surgical strategy. The optimal surgical strategy for RLPS, particularly WDLPS, is still unclear [3]. Inaccurate preoperative data may result in unnecessary resection of unaffected organs, heightened risks, and unfavorable prognoses.

Whole-body ^18^fluorine-fluorodeoxyglucose positron emission tomography-computed tomography (^18^F-FDG PET/CT) has shown its potential in the preoperative diagnosis and predicting biological behavior of RLPS [4–6]. However, relevant studies have focus solely on the maximum standardized uptake value (SUVmax). Furthermore, multiple studies have suggested that metabolic tumor volume (MTV) and total lesion glycolysis (TLG) can predict the prognosis of sarcomas of the extremities, [7–10] although no such study has been conducted for RLPS.

This study aims to assess the prognostic and pathological predictive capabilities of MTV, TLG, and SUVmax. Armed with the preoperative information provided by volume-based ^18^F-FDG PET/CT, we seek to investigate their potential impact on the surgical strategy and prognostic prediction of RLPS.

## Methods

### Patients

This study is a single-center retrospective study. A total of 89 patients with RLPS were included. These patients underwent surgery and had ^18^F-FDG-PET/CT scans at the Sarcoma Center of Peking University Cancer Hospital over a six-year period, from November 2013 to September 2019. The study included a consecutive series of patients. Prior to undergoing ^18^F-FDG-PET/CT scans, none of the patients had received any anti-tumor treatment, except for surgical resections. Ethical approval and written informed consent were obtained from all participants. Patient anonymity has been preserved. The histological grade of RLPS was reassessed based on the FNCLCC system by two experienced pathologists [11]. These pathologists were kept unaware of the ^18^F-FDG-PET/CT findings, as well as the clinical and prognostic information of the patients. A total of 17 patients were excluded from the prognostic analysis due to R2 resection and non-tumor-related causes of death.

### Inclusion and exclusion criteria

1. Patients whose preoperative diagnosis and postoperative pathology were RLPS would be included. Other patients were excluded.
2. Prior to undergoing ^18^F-FDG-PET/CT scans, none of the patients had received any anti-tumor treatment, except for surgical resections.
3. No distant metastasis was found before or during surgery.
4. All patients signed an informed consent form and agreed to participate in the study.
5. Patients with multiple primary tumors were excluded from the study.

### Acquisition of images with ^18^F-FDG PET/CT

Imaging was performed using a PET/CT scanner (Biograph64, SIEMENS, Erlangen, Germany) operated by 3D Flow Motion (bed entry speed 1 mm/s) from the apex of the skull to the mid-thigh. The PET axial field of view was 21.6 cm, and the images were reconstructed using the TrueX + TOF method. Low-dose CT scans were acquired in CARE Dose4D mode (120 kV, image slice thickness, 3.0 mm), and the patients were instructed to fast for at least six hours before ^18^F-FDG injection. In all cases, the serum glucose concentration met the institutional requirements (≤ 120 mg/dL). The injected activity was 3.7 MBq/kg, and the time from injection to scan was 60 min. We calculated SUV by normalization of FDG uptake to patient body weight (SUVbw).

**Fig. 1:**
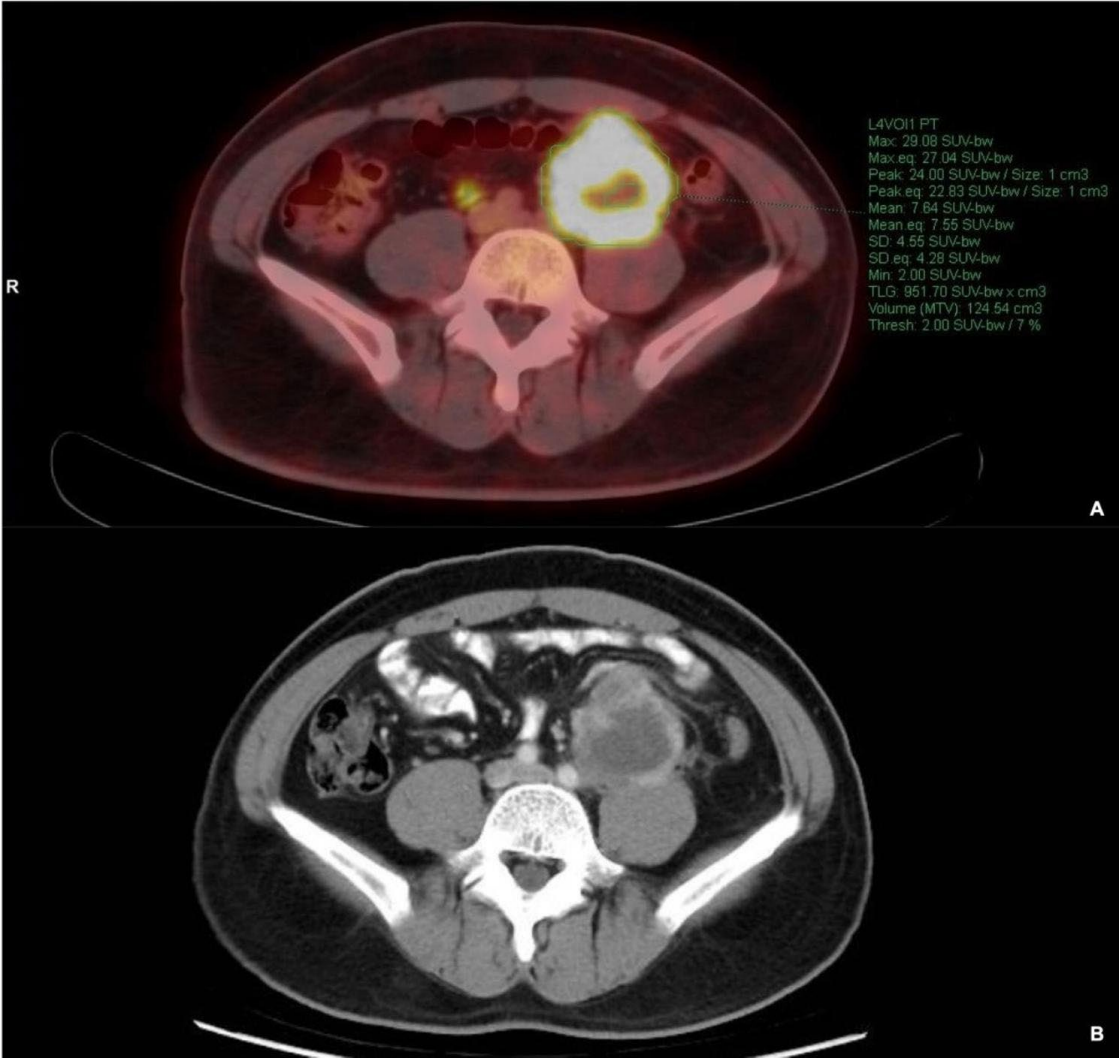
**A** and **B** illustrate examples of MTV and TLG measurement

A Siemens workstation (MultiModality WorkPlace, Erlangen, Germany) was used for the MTV and TLG measurements. The MTV of each lesion was calculated from the PET data using a semi-automated contouring program that allowed both an absolute and a relative threshold for segmentation. Human intervention was employed for necessary corrections to the contouring, particularly when physiological uptake was incorrectly delineated. In agreement with previous studies, we set an absolute SUVmax threshold of 2.0 for all patients [7]. Briefly, a tridimensional region of interest was drawn around each target lesion, and all spatially connected voxels with SUVmax ≥ 2.0 were grouped. Then, the volume of the delineated tumor was recorded, along with the SUVmax and SUVmean values (Fig. 1). The TLG of each lesion was calculated by multiplying the MTV by the corresponding value of SUVmean value. The MTV and TLG were calculated by the sum of the corresponding values whenever multiple lesions showed. MTV, TLG and SUVmax were measured by two experienced nuclear medicine technologists, who were blinded to the clinical, prognostic, and pathological findings.

### Statistics

Data collection and statistical analyses were performed using IBM SPSS Version 26 (SPSS Inc, Chicago, IL, USA). Enumeration data were expressed as mean and standard deviation and ranked data by cross-tabulation and percentages. A ROC curve analysis was employed to ascertain the optimal cut-off value of SUVmax, MTV, and TLG in discriminating between mortality and survival. Disease free survival (DFS) and overall survival (OS) were estimated by the Kaplan-Meier method. DFS and OS were calculated from date of surgery. The log-rank test was used to determine statistical differences in OS and DFS. For statistical analysis, the t-test, linear regression, Pearson correlation analysis, ANOVA, non-parametric test, chi-square test, and log–rank test were used. All tests were two-sided, with a statistical significance threshold of *P* ≤ 0.05.

## Results

### Pathological characteristics

SUVmax was associated with mitotic count and Ki-67 index (*P* < 0.001 and *P* = 0.024, respectively), whereas MTV and TLG were not (*P* = 0.282, *P *= 0.828, *P* = 0.118 and *P* = 0.606, respectively). SUVmax correlated with the histological grade of RLPS, and the SUVmax of G1, G2, and G3 were significantly differed (*P* for G1/G2 = 0.005, *P* for G2/G3 = 0.017, and *P* for G1/G3 = 0.001, respectively). Specifically, the SUVmax for G1, G2, and G3 were 3.02 ± 1.72, 7.24 ± 5.59, and 10.29 ± 8.30, respectively. MTV and TLG of G3 RLPS were significantly higher than G1 RLPS, whereas there were no significant differences between G1/G2 and G2/G3 (*P* for G1/G2 were 0.112 and 0.270, *P* for G2/G3 were 0.364 and 0.158, and *P* for G1/G3 were 0.001 and 0.010, respectively). MTV, TLG and SUVmax of WLPS were significantly lower than those of PLPS and DDLPS (*P* for MTV were 0.009 and 0.022, *P* for TLG were 0.028 and 0.048, and *P* for SUVmax were 0.027 and < 0.001, respectively). MTV, TLG and SUVmax showed no significant difference between PLPS and DDLPS (*P* = 0.755, *P* = 0.257 and *P* = 0.778, respectively).

### Clinical characteristics

The TLG and MTV of multifocal RLPS were significantly higher than those of unifocal RLPS (*P* < 0.001 and *P* < 0.001, respectively), whereas the SUVmax was not (*P* = 0.933). Recurrent RLPS showed a higher MTV and TLG than primary RLPS (*P* = 0.003 and *P* = 0.002, respectively), but there was no statistically significant difference in SUVmax (*P* = 0.797). MTV, TLG and SUVmax significantly correlated with tumor size (*P* = 0.001, *P* = 0.018 and *P* = 0.012; R = 0.334, R = 0.251 and R = 0.267, respectively). MTV, TLG and SUVmax did not correlated with number of pathological invasions of adjacent organs (*P* = 0.133, *P* = 0.055 and *P* = 0.694, respectively). MTV and TLG significantly correlated with blood loss (*P* = 0.012 and *P* = 0.016; R = 0.265 and R = 0.256, respectively), whereas SUVmax did not (*P* = 0.084). MTV, TLG and SUVmax did not correlate with number of organs resected (*P* = 0.642, *P* = 0.708 and *P* = 0.465, respectively).

### Prognostic characteristics

**Fig. 2:**
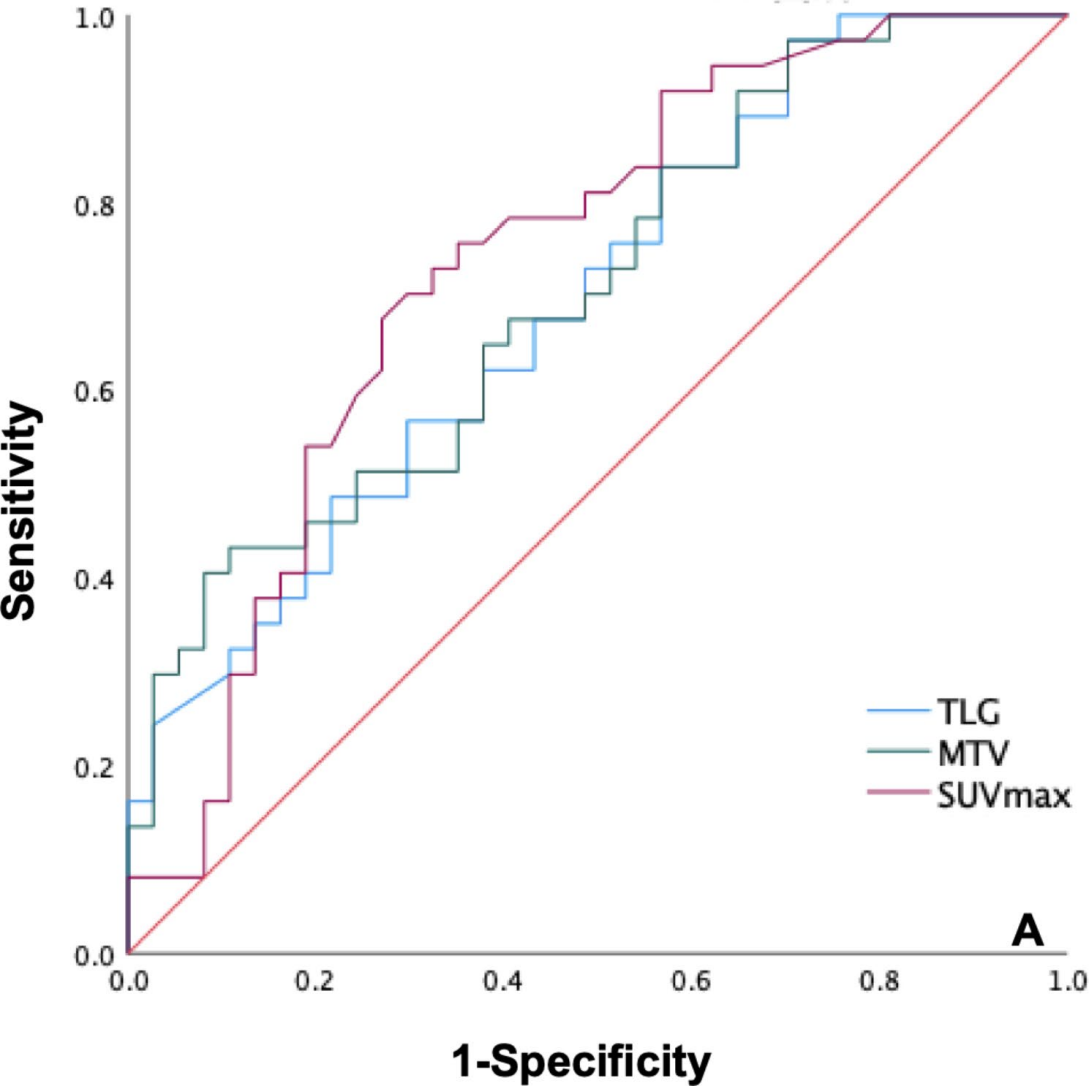
The figure shows the ROC curves for SUVmax, MTV, and TLG to between mortality and survival

**Fig. 3:**
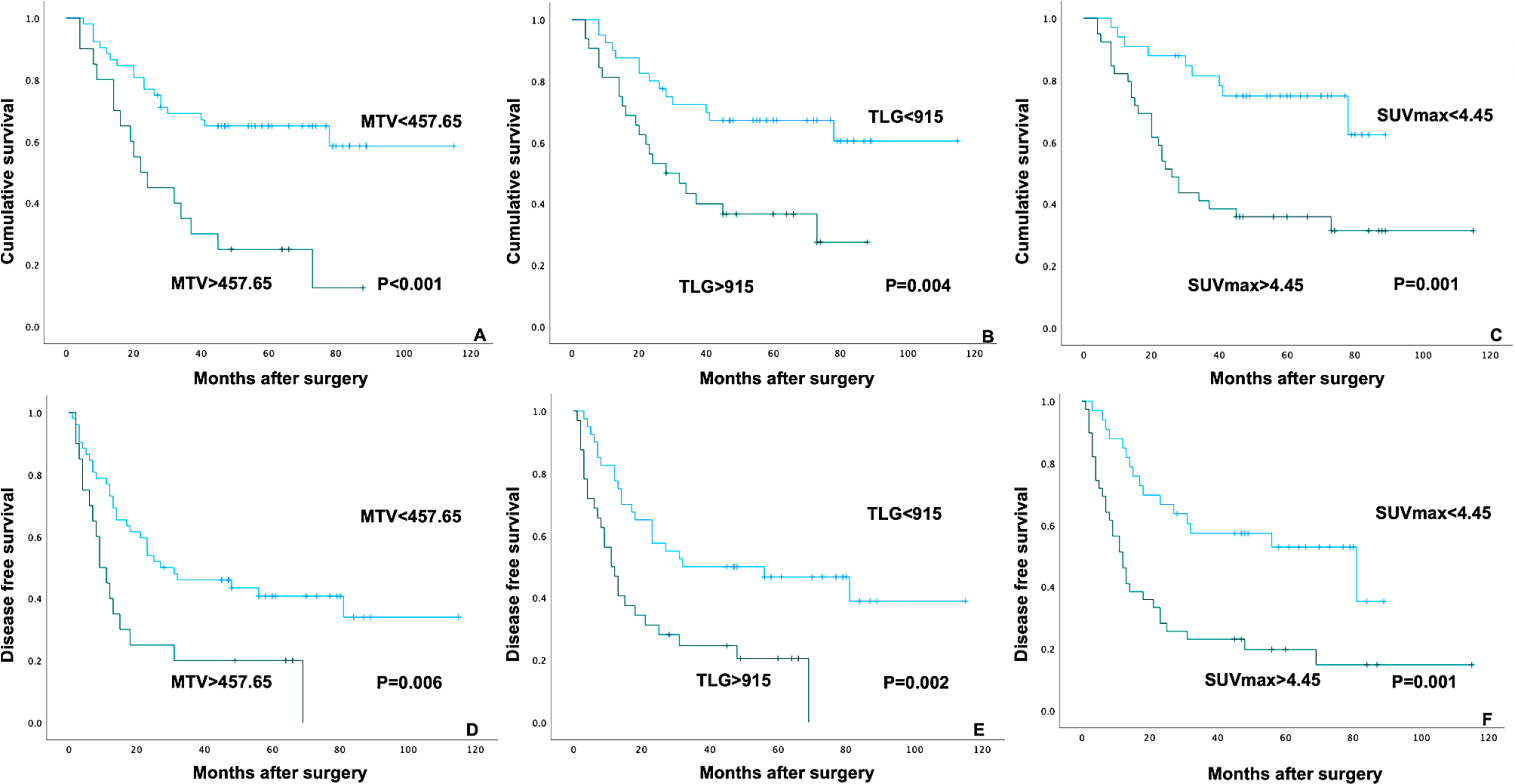
**A-F** shows the Kaplan–Meier survival curves for SUVmax, MTV, and TLG cutoff value

ROC curve analysis suggested 457.65, 915, and 4.45 as the optimal cutoff values of MTV, TLG and SUVmax in discriminating between mortality and survival (Fig. 2). MTV, TLG and SUVmax cut-off values showed statistically significant associations with OS and DFS using the Kaplan–Meier method (*P* for MTV were < 0.001 and 0.006, *P* for TLG were 0.004 and 0.002, and *P* for SUVmax were 0.001 and 0.001, respectively, Fig. 3). The clinical characteristics of patients are listed in Table-1. Multifocal RLPS, pathological subtype, recurrent RLPS and FNCLCC histological grade significantly correlated with DFS and OS using the Kaplan–Meier method (*P* for multifocal RLPS were 0.010 and 0.001, *P* for pathological subtype were 0.004 and 0.001, *P* for recurrent RLPS were 0.006 and < 0.001, and *P* for FNCLCC histological grade were < 0.001 and < 0.001, respectively). Tumor size did not correlate with OS and DFS (*P* = 0.460 and *P* = 0.205). Multivariable Cox regression analyses were performed for OS. Potential risk factors for death in the univariable analyses were used for multivariable adjusted models. Multivariable Cox analysis showed that MTV > 457.65 (*P* = 0.025), pathological subtype (*P* = 0.049) and FNCLCC histological grade (*P* = 0.033) were related to OS.

**Table 1:** Clinical characters of RLPS for prognostic analysis using cutoff value of MTV

Parameters		n	MTV > 457.65 (95% CI)	MTV < 456.65 (95% CI)	*P*-value	Odds Ratio (95% CI)
**Age(y)**			**54.85 ± 10.07** **(47.42, 58.21)**	**54.73 ± 10.48** **(52.40, 58.35)**	**0.784**	**—**
**Operative time (min)**			**606.10 ± 198.81** **(456.51, 657.86)**	**470.19 ± 163.69** **(429.57, 525.30)**	**0.083**	**—**
**Blood loss (ml)**			**3320.00 ± 2797.67** **(1182.93, 3867.07)**	**1799.23 ± 1869.47** **(1356.35, 2462.82)**	**0.014**	**—**
**Gender**					**0.612**	**1.31 (0.46–3.72)**
	**Male**	**43 (59.7%)**	**11 (15.3%)**	**32 (44.4%)**		
	**Female**	**29 (40.3%)**	**9 (12.5%)**	**20 (27.8%)**		
**Tumor size (cm)**			**29.68 ± 15.59 ** **(19.20, 35.37)**	**20.80 ± 9.93** **(18.00, 23.57)**	**0.141**	**—**
**Pathological subtype**					**0.069**	
	**WDLPS**	**17 (23.6%)**	**1 (1.4%)**	**16 (22.2%)**		**—**
	**DDLPS**	**44 (61.1%)**	**15 (29.4%)**	**29 (40.3%)**		**9.14 (0.86–97.27)**
	**PLPS**	**11 (15.3%)**	**4 (5.6%)**	**7 (9.7%)**		**8.28 (0.99–68.55)**
**Histological grade (FNCLCC)**					**0.24**	
	**G1**	**19 (26.4%)**	**3 (4.2%)**	**16 (22.2%)**		**—**
	**G2**	**33 (45.8%)**	**9 (12.5%)**	**24 (33.3%)**		**2.00 (0.47–8.54)**
	**G3**	**20 (27.8%)**	**8 (11.1%)**	**12 (16.7%)**		**3.56 (0.78–16.31)**
**Complication (Dindo–Clavien Classification)**					**0.635**	
	**I**	**31 (43.1%)**	**7 (9.7%)**	**24 (33.3%)**		**—**
	**II**	**29 (40.3%)**	**8 (11.1%)**	**21 (29.2%)**		**1.31 (0.41–4.21)**
	**IIIa**	**9 (12.5%)**	**4 (5.6%)**	**5 (6.9%)**		**2.74 (0.58–13.07)**
	**IIIb**	**3 (4.2%)**	**1 (1.4%)**	**2 (2.8%)**		**1.71 (0.14–21.82)**
**Length of stay (days)**			**37.00 ± 12.04** **(29.73, 42.90)**	**29.92 ± 15.43** **(25.74, 34.96)**	**0.664**	**—**
**Resection of organs**			**7.65 ± 3.84** **(5.32, 9.18)**	**5.90 ± 2.58** **(5.24, 6.76)**	**0.007**	**—**
**Multifocal disease**					**0.151**	**2.22 (0.74–6.70)**
	**Yes**	**20 (27.8%)**	**8 (11.1%)**	**12 (16.7%)**		
	**No**	**52 (72.2%)**	**12 (16.7%)**	**40 (55.6%)**		
**Vascular resection**					**0.863**	**1.11 (0.34–3.69)**
	**Yes**	**17 (23.9%)**	**5 (6.9%)**	**12 (16.7%)**		
	**No**	**55 (76.4%)**	**15 (20.8%)**	**40 (55.6%)**		
**Organs invaded by**			**2.19±1.72**	**1.08±1.09**	**0.159**	**–**
**sarcoma**			**(1.27, 3.10)**	**(0.77, 1.40)**		

## Discussion

Retroperitoneal liposarcoma (RLPS) presents a unique clinical challenge characterized by a low metastatic rate but a high propensity for local recurrence. These tumors have long been known for their resistance to conventional chemotherapy and radiotherapy, making surgical intervention the cornerstone of treatment. However, the approach to surgery must be highly individualized, taking into account both the characteristics of the tumor and the patient [12]. An inaccurate preoperative diagnosis can lead to inappropriate treatment strategies and unfavorable prognoses [3]. In our study, we have uncovered that volume-based ^18^F-FDG PET/CT scans offer valuable preoperative, noninvasive indicators for discerning RLPS pathological subtypes, histological grades, and prognostic outcomes, based on measurements of SUVmax, MTV, and TLG.

For the measurement of MTV and TLG in liposarcoma, there is currently no consensus on the optimal SUV threshold. Previous studies on sarcoma patients have indicated that an SUV threshold of 1.5 does not provide clear discrimination between the tumor and neighboring normal tissue, while an SUV threshold of 3 tends to underestimate the tumor area [13]. For WDLPS, the reported SUVmax values were 2.3 ± 1.2 and 2.8 (IQR: 1.9–3.2) in previous studies [4, 6]. Using a threshold SUV of 2.5 could also lead to a significant underestimation of the tumor area in RLPS. Therefore, in our study, we opted for a threshold SUV of 2 for MTV and TLG measurements.

Our study reveals that volume-based ^18^F-FDG PET/CT can effectively predict histological grade and multifocal disease while also distinguishing between WDLPS and DDLPS and PLPS. The primary objective of surgery for RLPS is complete resection, ideally as a single, intact specimen encompassing all involved contiguous organs. This typically necessitates en bloc resection of adjacent organs when there is obvious invasion, frequently involving the colon and kidney. Nonetheless, some proponents of extended resection argue for en bloc resection of adjacent organs that appear macroscopically uninvolved [3]. Critics of extended resection, however, express concerns regarding the high rate of multicentric disease and the location of recurrences. Extended resections in a substantial percentage of patients may not encompass potential multi-local disease recurrence and could only increase the morbidity of the procedure [12]. The extent of resection has been a subject of intense debate and should be guided by preoperative imaging. Pathological subtype, histological grade, and the presence of multifocal disease are the primary considerations in determining the surgical strategy for RLPS. Armed with preoperative information offered by volume-based ^18^F-FDG PET/CT, surgeons can make more precise and personalized decisions regarding the surgical approach.

Interestingly, we observed no significant correlation between MTV and TLG and the number of organ resections. This lack of correlation may be attributed to our aggressive surgical approach, wherein we performed an average of 6.57 ± 3.08 organ resections, despite the pathological invasion of adjacent organs being only 1.40 ± 1.39 on average. This suggests that preoperative information may not be sufficient in the decision-making process for RLPS surgical strategies. From an alternative perspective, we found that MTV and TLG do correlate with blood loss during surgery, implying that these metrics can offer some predictive value regarding operative difficulty. We hope that future data collection will yield significant association between different surgical data and MTV, SUVmax and TLG of RLPS, given the potential of volume-based ^18^F-FDG PET/CT to guide appropriate surgical strategies for RLPS.

**Fig. 4:**
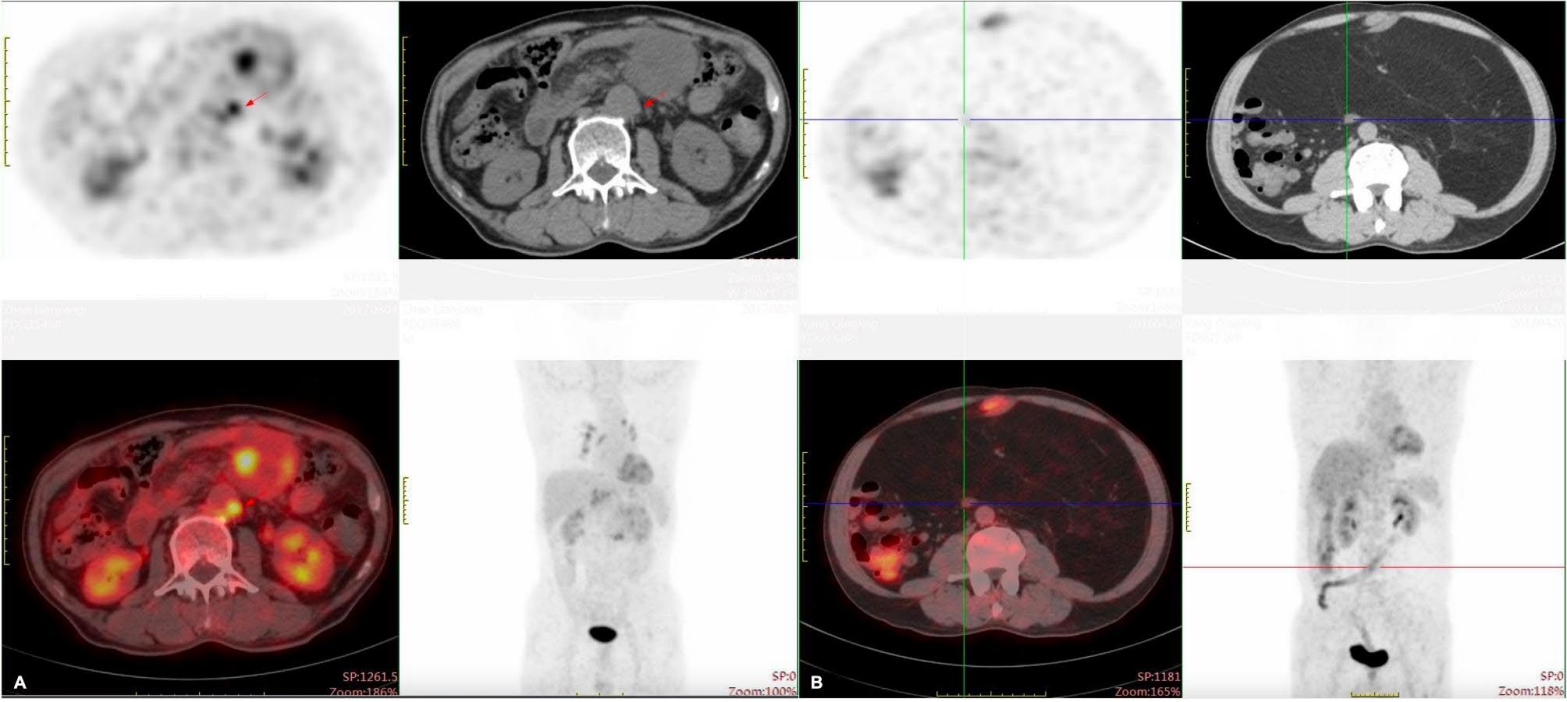
**A** shows a patient with non-recurrent PLPS with 9 cm diameter, 6.1 SUVmax, 618.2 MTV, and 1537 TLG in our cohort. The patient had 18 months DFS and 34 months OS. **B** shows a patient with non-recurrent WDLPS with 45 cm diameter, 1.4 SUVmax, 0 MTV, and 0 TLG in our cohort. The patient does not experience recurrence until the last follow-up, and its OS is 84 months

Univariable analysis demonstrated that we can predict the prognosis of RLPS using cutoff values for SUVmax, MTV, and TLG. The relationship between SUVmax and prognosis is rooted in the biology of RLPS, while MTV and TLG reflect the total volume and activity of metabolically active tumor cells. We observed that SUVmax correlates with histological grade, mitotic count, and Ki-67 index, while MTV and TLG correlate with the presence of multifocal disease. The combination of SUVmax, MTV, and TLG provides a more comprehensive approach to predicting the prognosis of RLPS, as it considers both the tumor biology and the metabolically active tumor volume. This prognostic prediction is particularly beneficial for patients with large tumor sizes and low values for SUVmax, MTV, and TLG (Fig. 4).

Multivariable Cox analysis revealed associations between MTV, pathological subtype, FNCLCC histological grade, and overall survival (OS). MTV holds promise as a new parameter for the development of an RLPS nomogram. Compared to traditional prognostic factors like age and tumor size, MTV is a preoperative, noninvasive, and reliable factor. We noted the absence of relationship between OS, DFS, and tumor size, which is traditionally considered a prognostic factor in the nomogram for retroperitoneal soft tissue sarcoma [14]. This phenomenon may be attributed to the impact of our aggressive surgical policies. As the utilization of these policies continues to rise, MTV could potentially surpass tumor size as a more valuable prognostic factor.

The study demonstrates that volume-based ^18^F-FDG PET/CT can provide preoperative, noninvasive markers for RLPS pathological subtypes, histological grades, biological behavior, and prognosis based on SUVmax, MTV, and TLG. However, challenges can arise when initially diagnosing a dedifferentiated component, and areas of potential dedifferentiation should be targeted for biopsy in these large tumors. It’s worth noting that percutaneous biopsy may miss the dedifferentiated component, with a false negative rate exceeding 50% in one study [15]. Guidance based on the “SUVmax location” may prove helpful for biopsy and pathological dissection.

It’s important to acknowledge the limitations of our study. Benign tumors such as lipoma and hibernoma were not included, preventing us from assessing the value of volume-based ^18^F-FDG PET/CT in determining surgical indications for retroperitoneal lipomatous tumors. Additionally, while volume-based ^18^F-FDG PET/CT offers valuable insights, it is not a comprehensive diagnostic tool, and information from other modalities such as CT, MRI, or biopsy remains essential for managing RLPS. Intraoperatively, liposarcoma can closely resemble normal fat, posing a challenge for those inexperienced with the disease. Volume-based ^18^F-FDG PET/CT may help predict the distribution of the tumor and normal fat, and our aim for future studies is to explore the use of ^18^F- FDG-PET/CT and ultrasound image fusion for improved diagnosis and treatment of RLPS.

## Conclusion

Our findings indicate that MTV is an independent prognostic factor for RLPS, while MTV, TLG, and SUVmax can preoperatively predict multifocal lesions, histological grade, and pathological subtype. Volume-based ^18^F-FDG PET/CT offers valuable information to aid in the decision-making process for RLPS surgical strategies.

### Electronic supplementary material

Below is the link to the electronic supplementary material.

Supplementary Material 1

## Data Availability

The datasets generated and analysed during the current study are not publicly available due consideration of medical ethics, but are available from the corresponding author on reasonable request.
