# Supplementary material for: Preoperative assessment of retroperitoneal Liposarcoma using volume-based 18F-FDG PET/CT: implications for surgical strategy and prognosis
Source: BMC Med Imaging. 2023 Dec 18;23:215. doi: 10.1186/s12880-023-01179-z (PMC10726537; doi:10.1186/s12880-023-01179-z)
Supplement: Supplementary file 1 — Supplementary Material 1 [file 12880_2023_1179_MOESM1_ESM.docx]

**Figure Legend**

**Supplementary Figure 1** The figure shows the flow chart of the study
